# Prognostic Factors for Low Visual Acuity after Cataract Surgery with Vitreous Loss

**DOI:** 10.1155/2021/6691904

**Published:** 2021-06-17

**Authors:** Michael Mimouni, Michal Schaap-Fogler, Philip Polkinghorne, Gilad Rabina, Rita Ehrlich

**Affiliations:** ^1^Department of Ophthalmology, Rambam Health Care Campus Affiliated with the Technion–Israel Institute of Technology, Haifa, Israel; ^2^Department of Ophthalmology, Rabin Medical Center, Petach Tikva, Israel; ^3^Department of Ophthalmology, University of Auckland, Auckland, New Zealand; ^4^Department of Ophthalmology, Sackler Faculty of Medicine, Tel Aviv Medical Center, Tel Aviv, Israel

## Abstract

**Purpose:**

The purpose of this study is to find prognostic factors associated with low visual acuity in patients experiencing vitreous loss during cataract surgery.

**Methods:**

A retrospective, noncomparative, interventional, case study of patients experiencing vitreous loss during cataract surgery. Data collected included demographics, best corrected visual acuity (BCVA), axial length (AL), presence of ocular comorbidity affecting central vision, timing of intraocular lens (IOL) implantation, position of the implanted lens, and the presence of corneal sutures. Low visual outcome was defined as BCVA < 20/40.

**Results:**

Overall, 179 patients (60.3% males) with a mean age of 73 ± 12 years and axial length of 23.5 ± 1.3 mm with a mean follow-up of 12 ± 13 months were included. In multivariable logistic regression analysis, low visual outcome was independently associated with persisting postoperative complications (OR 6.25, 95% CI 1.378–30.9), preexisting ocular comorbidities (OR 4.45, 95% CI 1.1–18.00), and secondary intraocular lens (IOL) implant (OR 10.36, 95% CI 1.8–60.00). Conversely, pars plana vitrectomy (PPV) for dislocated fragments of lens material, age > 70 years, gender, axial length, degree of surgeon, corneal suturing, and anterior chamber lens implantation were not found to have significant associations with low visual outcomes (*P* > 0.05).

**Conclusions:**

Low visual outcome after vitreous loss during cataract surgery was associated with ocular comorbidities, secondary IOL implantation, development of cystoid macular edema, and additional surgical complications.

## 1. Introduction

The frequency of vitreous loss during cataract surgery using phacoemulsification techniques ranges from 1.1% to 5% [1–8]. Risk factors for posterior capsular rupture that may lead to vitreous loss include small pupil, previous ocular trauma, pseudoexfoliation (PXF), poor visualization secondary to corneal opacities, corneal transplantation, postradial keratotomy, dense/mature cataract, zonular weakness, lack of patient cooperation, posterior polar cataract, postvitrectomy, and inherited conditions such as Marfan's syndrome and deep or shallow anterior chamber [2, 3, 9]. Intraoperative signs for posterior capsule rapture with vitreous loss may include sudden deepening of the anterior chamber, transitory appearance of a clear red reflex peripherally, inability to rotate a previously mobile nucleus, difficulty in burying the phaco needle into the nucleus, and tipping of one pole of the nucleus [10]. Vitreous loss can be associated with an adverse functional outcome secondary to either cystoid macular edema, corneal decompensation, glaucoma, endophthalmitis, or a combination thereof [11–13]. It is not clear what variables may impact the final postoperative acuity following this complication [14]. Some reports associate preexisting ocular comorbidities and advancing years with a low functional outcome [3]. Equally, a low preoperative acuity, the presence of age-related macular degeneration (AMD), and the need for a secondary pars plana vitrectomy (PPV) for dislocated lens material are reported to have a negative effect on acuity [3, 15].

The aim of this study is to try and determine what factors are associated with a low visual acuity in patients experiencing vitreous loss during cataract surgery.

## 2. Methods

This study was approved by the Institutional Review Board of the University of Auckland, Auckland, New Zealand. A retrospective study was conducted of all patients who underwent phacoemulsification surgery complicated by vitreous loss from 2004 through 2010 at the University of Auckland, Auckland, New Zealand. All surgeries were performed by one of the four consultants or four trainees during that period. All of the consultants and trainees had previously performed over 500 operations and 50 surgeries, respectively. Surgeries performed by a trainee were all supervised by a consultant and if a complication was encountered, the consultant guided the trainee or took over the operation to provide the patient with a guarded operation. Medical records from the hospital computer database include intraoperative complications, and this vehicle was used to identify this cohort.

### 2.1. Patient Data

Preoperative parameters recorded included sex, age, best corrected visual acuity (BCVA), and axial length (AL). The presence or absence of ocular comorbidity affecting central vision was noted (e.g., age-related macular degeneration (*n* = 16), glaucoma (*n* = 15), proliferative diabetic retinopathy (*n* = 7), and cystoid macular edema (*n* = 7)). Intraoperative data included timing of intraocular lens (IOL) implantation, position of the implanted lens, and the presence or absence of corneal sutures. Additional data included was the surgeon grade, with surgeries performed by a consultant surgeon, a fellow, or a registrar. Postoperative follow-up time, final BCVA, and any subsequent surgical intervention were recorded. Low visual outcome was defined as BCVA < 20/40.

### 2.2. Statistical Analysis

All statistical analyses were performed with SPSS version 21.0. Chi-square was used to compare proportions and student's T test to compare means. Any potential confounder that had at least a modest (*P* < 0.20) relation with both the outcome variable and the predictor of interest was included in multivariate model analyses. Statistical significance was defined as *P* ≤ 0.05.

## 3. Results

Overall, 179 cases of vitreous loss during cataract surgery using phacoemulsification were identified. Mean age and axial length were 73 ± 12 years and 23.5 ± 1.3 mm. There were 60.3% males (*n* = 108) and the mean follow-up time was 12.4 ± 13.4 months (range: 1–60 months).

Risk factors for capsular rupture were identified in 30% (*n* = 54) with the most common being dense/mature cataract (*n* = 33). Additional factors were narrow pupils, pseudoexfoliation glaucoma, zonular weakness, posterior polar cataract, and lack of patient cooperation.

There was a significant improvement in BCVA from 1.13 ± 0.83 to 0.32 ± 0.43 logMAR at last follow-up (*P* < 0.05) with 82% ≥ 20/40. BCVA was reduced by 1 or more lines in 8.4% (*n* = 15) and by 2 or more lines in 5.6% (*n* = 10).

### 3.1. Visual Acuity and Preexisting Features

Age, sex, BCVA, and axial length were not found to have significant associations with low visual outcome (*P* ≥ 0.05). Ocular comorbidity was the only preoperative parameter significantly associated with low visual outcome in univariate analysis. Ocular comorbidity was present in 36% (*n* = 63). Patients undergoing cataract surgery with additional pathologies were 4 times more likely to have low visual outcome. In 31% of eyes with ocular comorbidity, final BCVA was worse than 20/40 compared to 7.7% in eyes without comorbidity. There was no significant association between specific comorbidities (glaucoma, AMD, and proliferative diabetic retinopathy) and low visual outcome.

### 3.2. Visual Acuity and Intraoperative Parameters

Secondary IOL implantation and the use of corneal sutures during surgery were associated with a low visual outcome (Table 1).

The IOL was positioned in the sulcus in 74% (*n* = 132), in the bag in 19% (*n* = 34), and in the anterior chamber in 7% (*n* = 1). Twelve of the eyes where the lens was implanted in the bag had a capsular tension ring inserted. Surgeon category was not associated with low visual outcome, with 59% eye (*n* = 106) operated by a consultant, 11% (*n* = 20) by a fellow, and 30% (*n* = 53) by a registrar.

### 3.3. Visual Acuity and Postoperative Parameters

Postoperative CME (7.9%, *n* = 12) increased the risk for low visual outcome by nearly 3 times (*P*=0.034). Other complications included increased intraocular pressure (*n* = 9), mild ERM (*n* = 4), and retinal detachment (*n* = 3).

Following primary surgery, 12 eyes were noted to be vitreous in the anterior chamber directed towards the cataract section. Treatment included Nd : YAG laser (*n* = 9) and surgical intervention (*n* = 3).

Overall, 22 eyes required additional surgery. Reasons included retained lens material (*n* = 14), IOL repositioning (*n* = 4), anterior vitrectomy (*n* = 3), and iris prolapse (*n* = 1).

The median interval between cataract surgery and PPV for retained lens material was one day (range: 0 to 60 days), with all but two patients operated within the first week. Data regarding patients that required PPV are summarized in Table 2. Final BCVA of 0.56 (±0.9) logMAR in eyes that required PPV for fragment loss was not significantly different from those not requiring PPV (*P*=0.09).

### 3.4. Multivariate Analysis

Table 3 depicts the results of the multivariate analysis. Preoperative ocular comorbidity, secondary IOL implantation and type of implanted lens, corneal sutures, secondary PPV, CME, and other surgical complications were included as predictors of low visual outcome. Preoperative ocular comorbidity, secondary IOL implantation, CME, and other surgical complications were associated with low visual outcome in multivariate analysis.

## 4. Discussion

This study and others [1, 2, 6, 16–18] clearly demonstrating vitreous loss during cataract surgery can be associated with a low functional outcome. Equally, some patients experiencing this complication can achieve excellent final acuities. The percentage of eyes achieving BCVA of 20/40 or better in the current series is similar to the previous reports, with 90% of eyes without ocular comorbidity and 68% with ocular comorbidity in our study achieving vision of 20/40 or better [2]. During follow-up, only 8.4% of patients had reduced visual acuity compared to their visual acuity before cataract surgery. This result is also comparable to the previous studies [2].

Surgically demanding cases are at increased risk for capsular rupture. These include small pupils, dense/mature cataract, instillation of trypan blue, and loose zonules with or without pseudoexfoliation [19]. In our study, 30% of the eyes experiencing capsular rupture during surgery were noted to have at least one of these risk factors, with dense/mature cataract as the leading cause.

Identifying factors influencing final visual acuity results, in eyes experiencing capsular rupture with and without vitreous loss, has been the purpose of the previous studies (Table 4). Low preoperative vision was found by Konstantopoulos et al. [15] to have a negative influence on visual results. Their rationale for this influence was that eyes with worse visual acuity had less visual potential as well as denser cataracts with increased operative difficulty. In our study, though there was a trend towards worst outcomes in patients with lower preoperative vision (OR = 1.89, *P*=0.03), it was both not significant and clinically negligible when compared to the presence of an ocular comorbidity (OR = 4.03, *P*=0.001). As such, though eyes with low preoperative visual acuity may have a tendency towards a more complicated surgery and perhaps worse outcomes, this is not always the case. Some will have a thick posterior subcapsular cataract which does not increase the challenge of such an operation and explains the very low vision. Therefore, we speculate that the preoperative visual acuity is not as good of an indicator of outcomes when compared to a preexisting ocular comorbidity.

As in previous studies, ocular comorbidity and CME were found to be associated with low visual outcome. Shah et al. found glaucoma but not diabetes was an adverse indicator while Konstantantopoulos et al. [15] found AMD impacted on the final acuity. Other authors have concluded ocular comorbidity has a negative effect, but without elaborating on how these factors might result in a negative outcome. Similarly, after assessing patients with glaucoma, AMD, and proliferative diabetic retinopathy, we could not isolate a specific eye condition predictive of an adverse outcome.

The optimal timing for a PPV to remove retained lens material in the vitreous is still under debate [13, 18–20]. Previous reports have shown that PPV performed within a week after the first operation may yield good final visual results, with as many as 70% of patients achieving final visual outcome of 20/40 or better. Others show that only delaying PPV beyond the first week after the complicated cataract surgery may compromise the final visual acuity results [18, 19]. In our study, 14 patients required PPV for this complication. Only 3 of these surgeries were performed during the primary procedure. Some reports suggest this might be the preferred technique and improve outcomes, but in our series, the numbers are too small to make a meaningful comment [13]. Furthermore, the logistics of having a vitreoretinal surgeon immediately available is not practical for most centers. In any event, we did not identify PPV as a statistically significant factor for an adverse outcome and could not identify the preferred time to intervene.

In the case of a posterior capsular rupture with vitreous loss, the IOL can be placed in front of the anterior capsule if the rhexis is round and the rim is adequate in size. But if the support is less than adequate, it may be necessary to place an anterior chamber IOL, iris fixated IOL, or scleral fixated IOL [20]. In cases of iris loss or aniridia, Kumar et al. demonstrated that glued aniridia IOL present good functional and anatomical results [21]. In this study, a secondary IOL implantation was associated with worse visual outcomes (OR = 10.36, *P*=0.009). To the best of our knowledge, other studies have not assessed the association of this factor with visual outcomes following vitreous loss during cataract surgery. We speculate that this may stem from two different causes. One, cases where an IOL was not implanted during primary surgery were probably more complicated to begin with than those in which an IOL was implanted. Two, it may be that additional surgery may expose the eye to further complications which can be avoided if the IOL is implanted during primary surgery.

Postoperative endophthalmitis is a serious complication of cataract surgery, with the incidence varying from 0.02% to 0.71%. Soliman et al. published a case series of 237 eyes with acute endophthalmitis secondary to cataract surgery, secondary lens implantation, or intravitreal injections. They concluded that pars plana vitrectomy was frequently performed regardless of the presenting vision in eyes with endophthalmitis and that increased vitreous opacification was associated with a higher probability for performing PPV [22].

This study has several limitations, first of which is its retrospective nature. Additional factors such as narrow pupils, pseudoexfoliation glaucoma, zonular weakness, and lack of patient cooperation were not considered in the analyses. Future, prospective studies may consider meticulously recording and thereafter analyzing these data. Second, results of surgeries from several surgeons were included in this study (*n* = 8). Future studies may consider analyzing the outcomes of a single surgeon to focus on the best strategy to achieve the best visual outcomes following vitreous loss.

## 5. Conclusions

This study recognizes ocular comorbidity, secondary IOL implantation, and subsequent postoperative CME as risk factors for low visual outcome in patients experiencing vitreous loss during cataract surgery. However, most patients with this complication do have good outcomes, especially if no comorbidities are present at the time of surgery. Similarly, patients requiring PPV for retained lens material can have good visual results although the timing of this additional intervention is not defined. Ophthalmologists, specifically cataract surgeons, should be aware of these findings in order to give their patients more accurate prognosis.

## Figures and Tables

**Table 1:** Univariate analysis of potential parameters associated with low visual outcome.

Parameter	Odds ratio (95% CI)	*P* value	Odds ratio (95% CI)
Age > 70 years	1.475 (0.6–3.3)	0.35	1.475 (0.6–3.3)
Female sex	1.05 (0.5–2.3)	0.9	1.05 (0.5–2.3)
Axial length (mm)			
Normal (22.01–24.99)	Referent		Referent
<normal	0.4 (0.1–2.5)	0.55	0.4 (0.1–2.5)
>normal	0.64 (0.7–5)	0.6	0.64 (0.7–5)
Preoperative BCVA < 20/40	1.886 (0.5–6.7)	0.3	1.886 (0.5–6.7)
Preoperative ocular comorbidity	4.03 (0.5–6.7)	0.001	4.03 (0.5–6.7)
Glaucoma	1.8 (0.5–5.9)	0.4	1.8 (0.5–5.9)
Age-related macular degeneration	2.8 (0.9–9.1)	0.6	2.8 (0.9–9.1)
Proliferative diabetic retinopathy	2.2 (0.9–9.1)	0.8	2.2 (0.9–9.1)
Surgeon rank (consultant as opposed to trainee)	0.8 (0.2–3.1)	0.8	0.8 (0.2–3.1)
Secondary IOL implantation	10.36 (1.8–60)	0.009	10.36 (1.8–60)
Location of implanted IOL (AC IOL as opposed to PC IOL)	2.854 (0.8–10)	0.11	2.854 (0.8–10)
Corneal sutures	0.3 (0.1–0.8)	0.02	0.3 (0.1–0.8)
Secondary pars plana vitrectomy	0.27 (0.1–0.7)	0.007	0.27 (0.1–0.7)
Cystoid macular edema	2.96 (1.1–8.1)	0.034	2.96 (1.1–8.1)
Other surgical complications	3.78 (1.7–8.4)	0.001	3.78 (1.7–8.4)

BCVA, best corrected visual acuity; IOL, intraocular lens; PC, posterior chamber; AC, anterior chamber.

**Table 2:** Details of eyes that required pars plana vitrectomy (PPV) for fragment loss.

Case	Preoperative BCVA	Preoperative ocular comorbidity	Timing of PPV after cataract surgery (days)	Postoperative BCVA	Postoperative complications
1	20/100	None	1	20/20	Increased IOP, required TRAB; further lens exchange
2	20/80	None	1	20/40	Intraocular lens decentration
3	20/63	NPDR	25	20/40	Mild ERM
4	20/40	None	1	20/25	None
5	3/63	None	60	20/25	Corneal edema
6	20/160	RRD repair	0	LP	Vitreous hemorrhage
7	20/20	None	1	20/20	None
8	LP	None	0	20/25	Retinal tear
9	20/80	None	6	20/32	None
10	20/80	PDR	4	20/32	None
11	3/63	None	1	20/20	None
12	20/160	Monocular diplopia	0	20/80	Neurotrophic cornea
13	20/63	None	3	LP	PVR
14	CF	None	0	20/160	RRD

BCVA, best corrected visual acuity; IOP, intraocular pressure; TRAB, trabeculectomy; NPDR, nonproliferative diabetic retinopathy; ERM, epiretinal membrane; RRD, rhegmatogenous retinal detachment; PDR, proliferative diabetic retinopathy; PVR, proliferative vitreoretinopathy.

**Table 3:** Multivariable analysis potential parameters associated with low visual outcome.

Parameter	Odds ratio (95% CI)	*P* value
Preoperative ocular comorbidity	4.4 (1.1–18)	0.03
Secondary IOL implantation	10.36 (1.8–60)	0.009
Implanted lens (PC IOL as opposed to AS IOL)	0.3 (0.3–3)	0.3
Corneal sutures	0.35 (0.7–1.8)	0.2
Secondary pars plana vitrectomy	3.5 (0.6–19)	0.15
Cystoid macular edema	2.96 (1.1–8.1)	0.034
Other surgical complications	6.5 (1.4–30)	0.02

IOL, intraocular lens; PC, posterior chamber; AC, anterior chamber.

**Table 4:** Previous reports on prognostic factors for visual acuity outcome following vitreous loss during cataract surgery.

No.	Authors	No. of eyes	Age (years)	Mean follow-up time	Final BCVA ≥ 20/40 (%)	Factors found to decrease final BCVA
1	Schaap-Fogler et al. (present study)	179 vitreous losses	73 ± 12	12 weeks	82	Univariate analysis: ocular comorbidity, secondary IOL implantation, corneal sutures, and CME; multivariate analysis: preoperative ocular comorbidity, secondary IOL implantation, CME, and other surgical complications
2	Meduri et al. [9]	230 vitreous losses	78.4 ± 11	13.4 weeks	53.9	Univariate analysis: preoperational BCVA, older age, ocular comorbidity, AMD, AL ≤ 22, AL ≥ 25, and secondary PPV; multivariate analysis: preoperational BCVA, AMD, and CME
3	Scott et al. [5]	142 capsular ruptures	67.6 (36–91)	>6 weeks	70.4	Univariate analysis: older age, ocular comorbidity, ECCE, AC IOL implantation, and need for anterior vitrectomy; multivariate analysis: older age, ocular comorbidity, and AC IOL implantation
4	Hashemi et al. [8]	92 vitreous losses	NA	NA	86	CME and retinal detachment
5	Hashemi et al. [7]	59 capsular ruptures	NA	19.3 weeks	54	Ocular comorbidity (incidental finding: older age)
6	Thevi et al. [4]	887 capsular ruptures	67 ± 10.4	1–3 months	93.8	Age > 65 years, dropped nuclei, and postoperative retinal, corneal, and IOL complications

BCVA, best corrected visual acuity; CME, cystoid macular edema; AMD, age-related macular degeneration; AL, axial length; ECCE, extracapsular cataract extraction; AC, anterior chamber; NA, information not available; IOL, intraocular lens.

## Data Availability

The data used to support the findings of this study are included within the article.
